# Supplementary figures and images for: Kar4, the yeast homolog of METTL14, is required for mRNA m6A methylation and meiosis
Source: PLoS Genet. 2023 Aug 21;19(8):e1010896. doi: 10.1371/journal.pgen.1010896 (PMC10470960; doi:10.1371/journal.pgen.1010896)

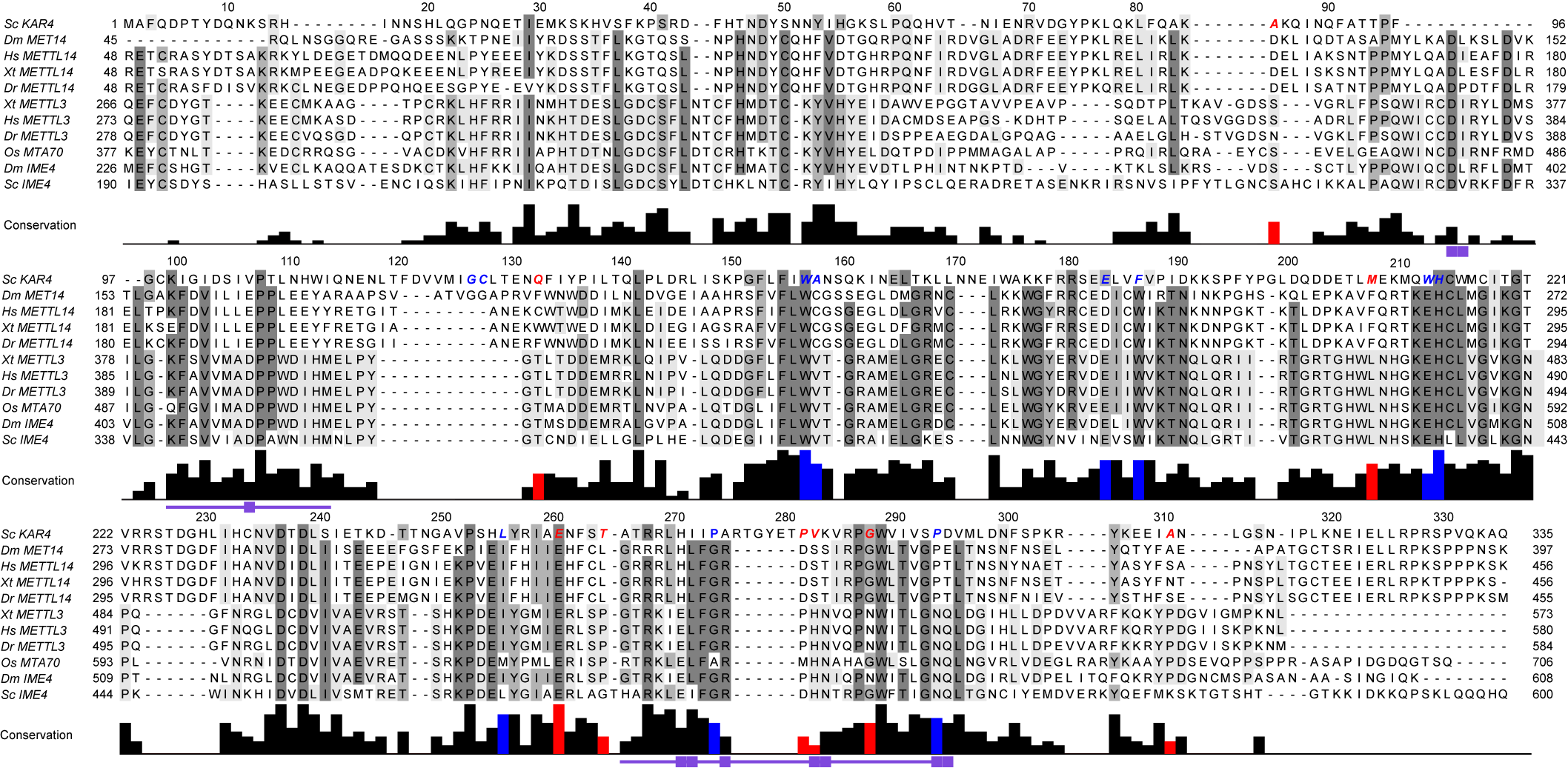

Supplement: S1 Fig — Sequence conservation of KAR4 and IME4 and a broad set of orthologs from a range of vertebrates, invertebrates, and plants: Sc, Saccharomyces cerevisiae; Dm, Drosophila melanogaster; Hs, Homo sapiens; Dr, Danio rerio; Xt, Xenopus tropicalis; Os; Oryza sativa. The degree of conservation is indicated by the height of the histogram below the sequence and by the shade of the background. Note the presence of blocks that are specific to Kar4p or Ime4p orthologues (light grey), as well as regions conserved between both paralogs (dark grey). Alleles defective for mating are in blue and alleles defective for meiosis are in red. Functional motifs associated with the methyltransferase activity of Ime4p orthologues [19] are indicated by the purple regions below the sequence. (TIF) [file pgen.1010896.s001.tif]

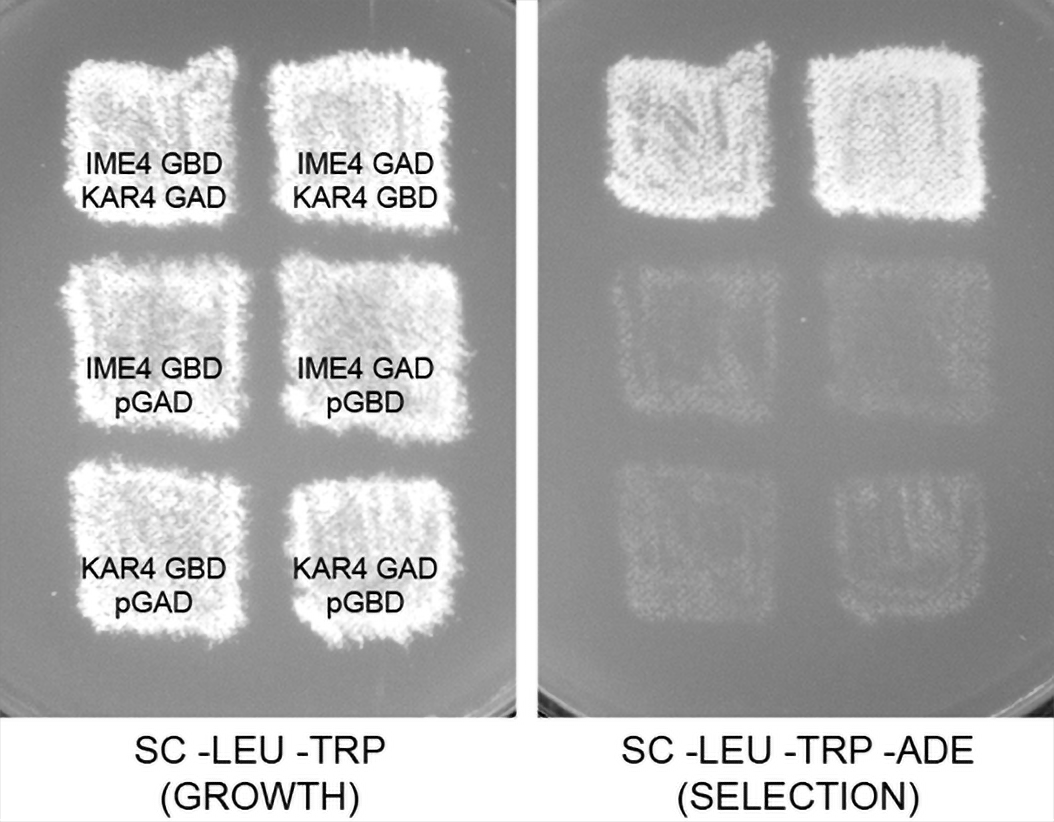

Supplement: S2 Fig — The combination of IME4 and KAR4 fusions was sufficient to drive the reporter gene (right), regardless of the orientation of the proteins fused to each hybrid. No fusion protein could activate the ADE2 reporter alone. All the strains grew on non-selective media (left). (TIF) [file pgen.1010896.s002.tif]

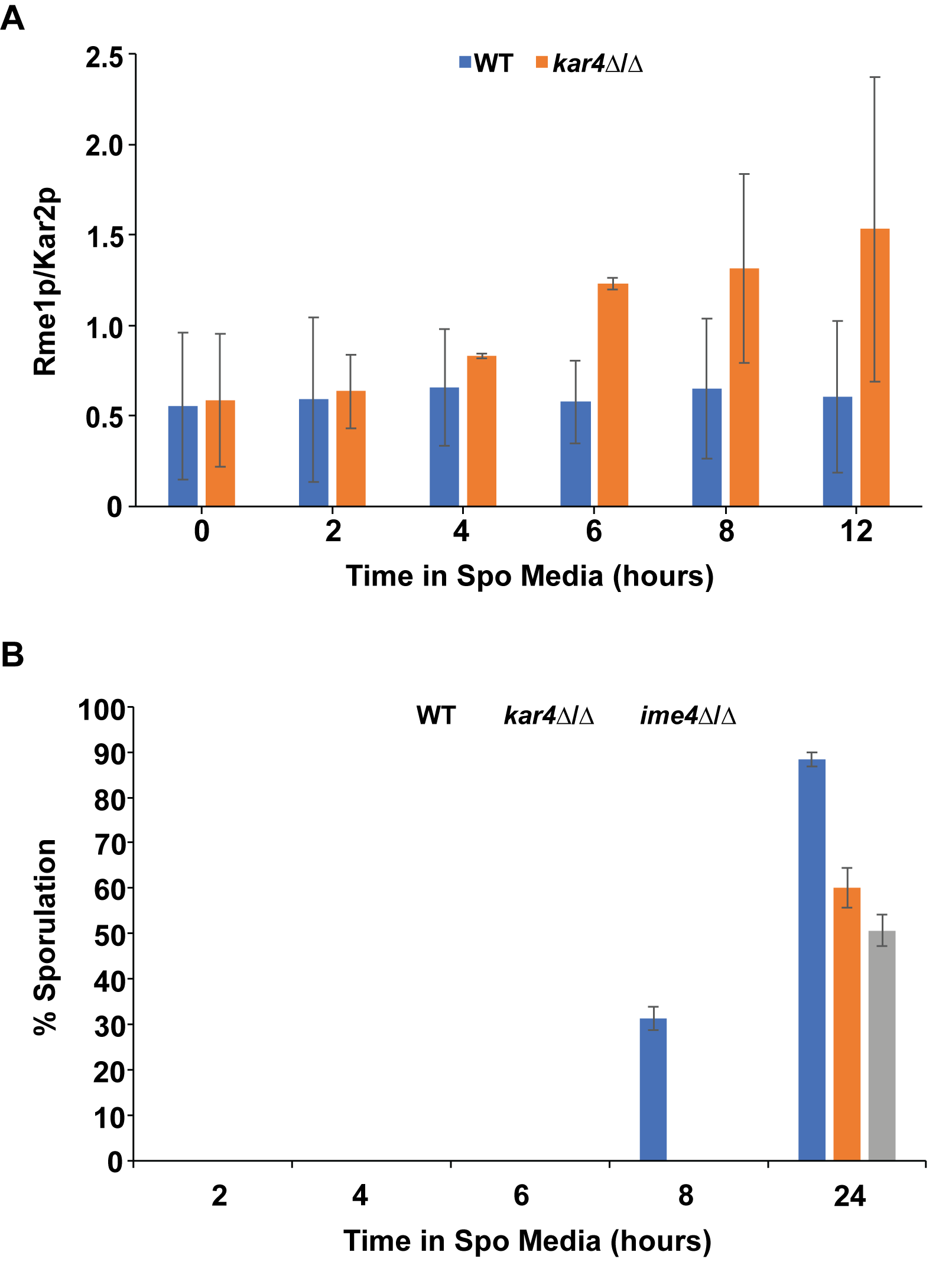

Supplement: S3 Fig — (A) Quantification of western blots from Fig 4B shown as the ratio of Rme1p to the loading control Kar2p. Error bars represent standard error (1/2 x the range) of two biological replicates. (B) Sporulation of wild type (MY 16325), kar4Δ/Δ (MY 16351), and ime4Δ/Δ (MY 16326) in the SK1 strain background. Colors are as in panel A; ime4Δ/Δ is in grey. All dyads, triads, and tetrads were counted, and 100 cells were counted for each time point. Error bars represent the standard deviation of three biological replicates. (TIF) [file pgen.1010896.s003.tif]

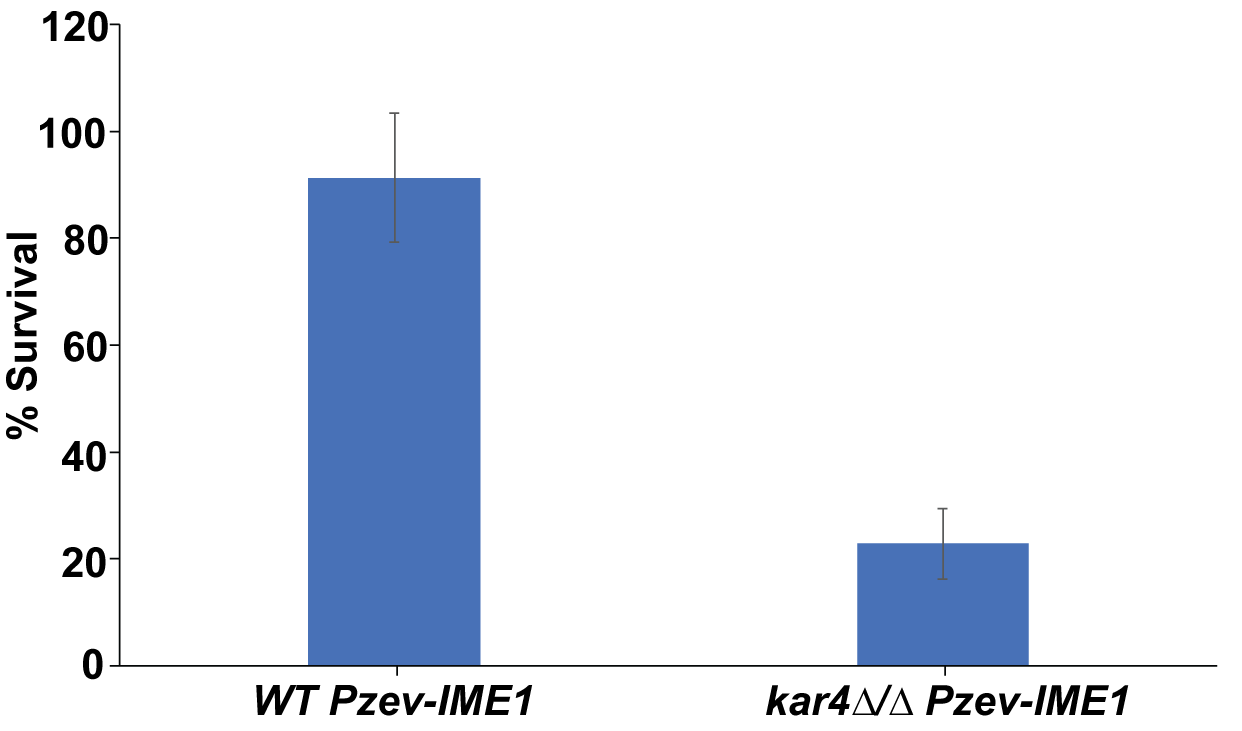

Supplement: S4 Fig — Viability of wild type (MY 16534) kar4Δ/Δ (MY 16531) mutants 48 hours after induction of IME1 expression in sporulation conditions. Viability was assessed as the change in colony forming units between t = 0 (before IME1 induction) and t = 48 hours after IME1 induction. Error bars represent the standard deviation of three biological replicates. (TIF) [file pgen.1010896.s004.tif]

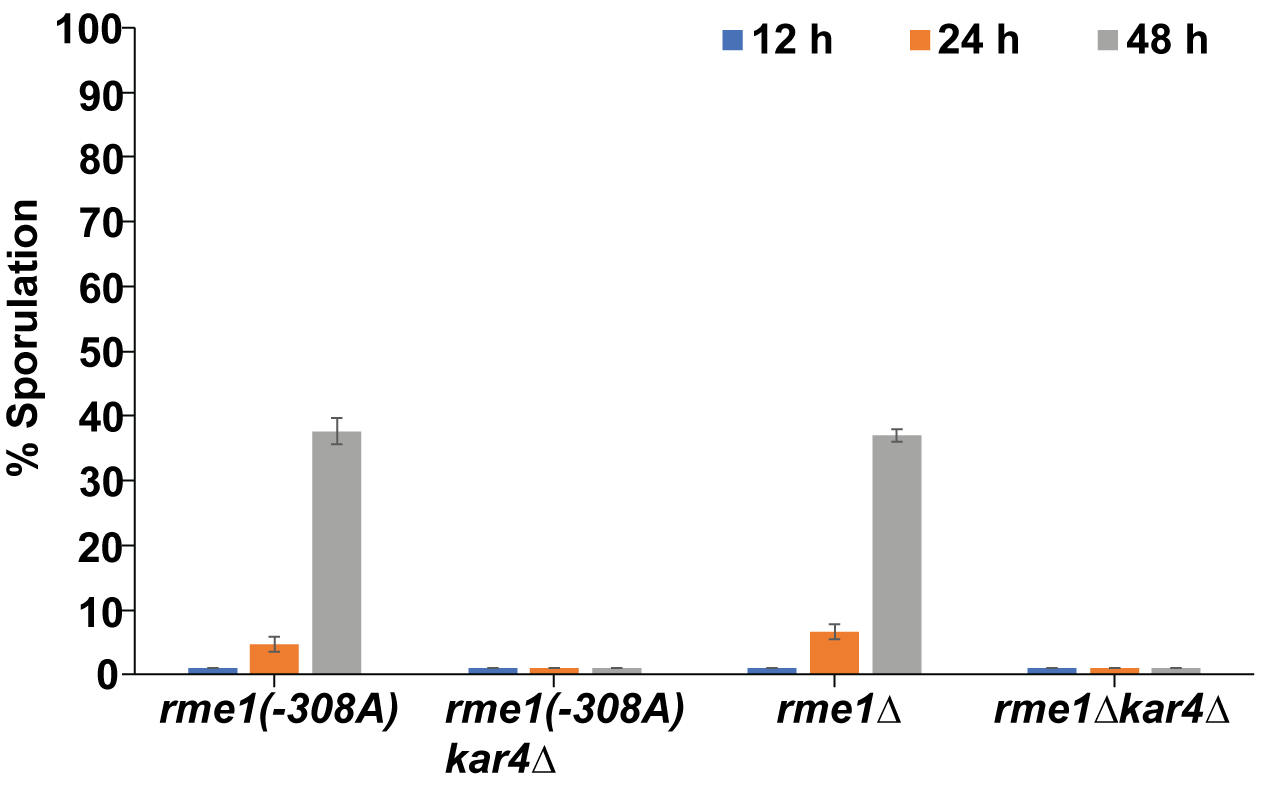

Supplement: S5 Fig — Spores were counted at the indicated time points in kar4Δrme1Δ/ kar4Δrme1Δ (MY 16566), kar4Δrme1(-308A)/ kar4Δrme1(-308A) (MY 16559), and the single mutants of each RME1 allele (MY 16456 and 16557). All dyads, triads, and tetrads were counted, and 100 cells were counted for each time point. Error bars represent the standard deviation of three biological replicates. (TIF) [file pgen.1010896.s005.tif]

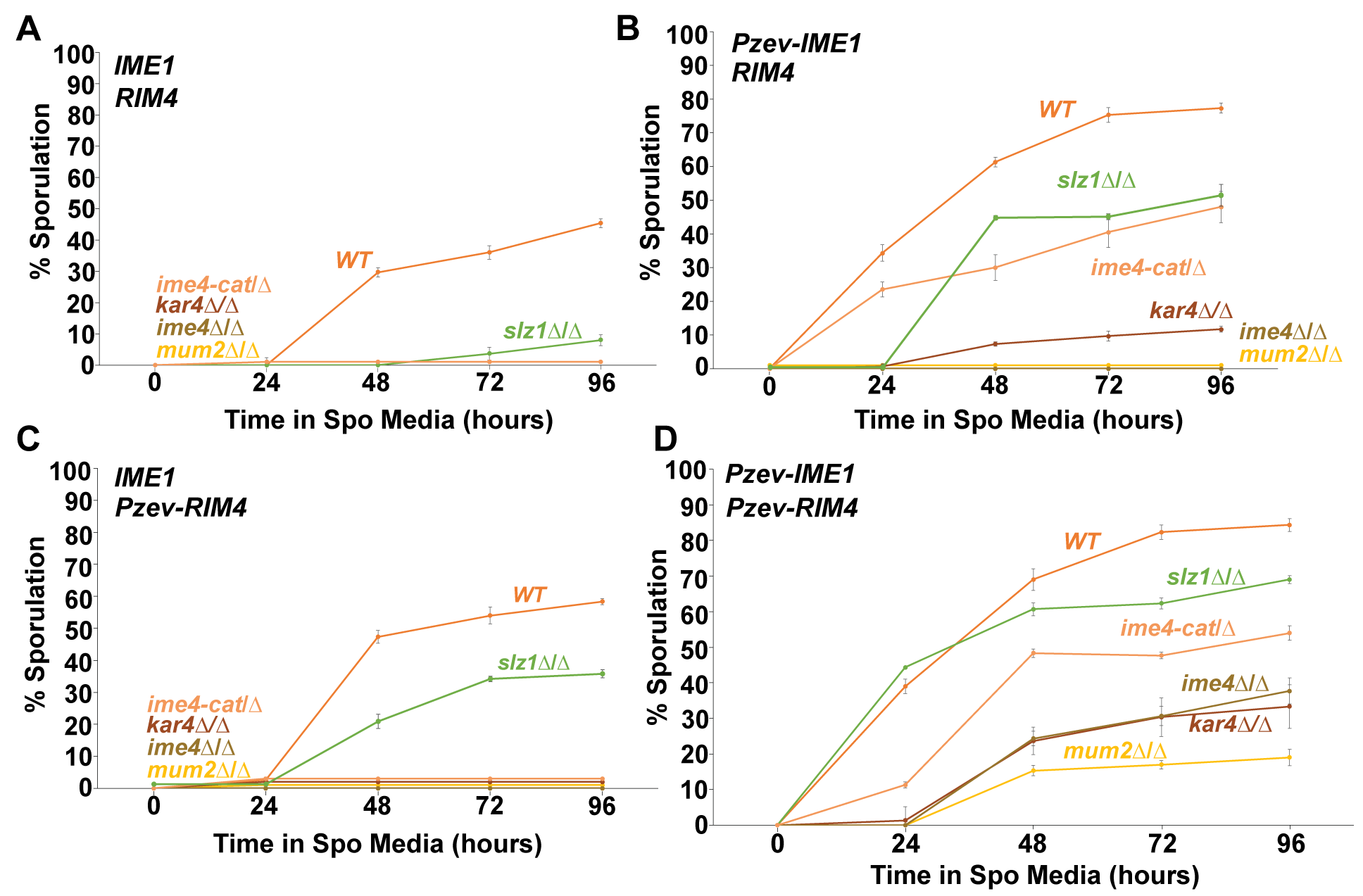

Supplement: S6 Fig — (A) Spore counts for the indicated mutants in IME1 RIM4 strains. (B) Spore counts for the indicated mutants in Pzev-IME1 RIM4 strains. (C) Spore counts for the indicated mutants in IME1 Pzev-RIM4 strains. (D) Spore counts for the indicated mutants in Pzev-IME1 Pzev-RIM4 strains. For each experiment all dyads, triads, and tetrads were counted, and 100 cells were counted at the indicated times. For experiments involving overexpression (B, C, and D), 1 μM of estradiol was used to induce expression. Strains are the same as those listed in the caption for Fig 8. (TIF) [file pgen.1010896.s006.tif]
