# Supplementary material for: Kar4, the yeast homolog of METTL14, is required for mRNA m6A methylation and meiosis
Source: PLoS Genet. 2023 Aug 21;19(8):e1010896. doi: 10.1371/journal.pgen.1010896 (PMC10470960; doi:10.1371/journal.pgen.1010896)
Supplement: S1 Table — All auxotrophic markers are standard BY alleles. All strains were constructed in this study unless otherwise noted. (DOCX) [file pgen.1010896.s007.docx]

| **Table S1. Strains used for this study. All auxotrophic markers are standard BY alleles.** | | | |
| --- | --- | --- | --- |
| Strain Name | Genotype | Strain | Reference |
| MY 10128 | *leu2 his3 ura3 met15 kar4::KanMX* | S288c |  |
| MY 11297 | *ura3 leu2 his3 lys2 can1::LEU2+-MFA1pr-HIS3 kar4::KanMX* | S288c |  |
| MY 16294 | *URA3::GFP-TUB1/+ SPC42-mcherry::HIS3/+ +/kar4::URA3 HPH::Pz3ev-IME1/+ leu2::ACT1PR-3ZEV-NatMX/leu2 his3/+ ura3/" met15/+* | S288c |  |
| MY 16295 | *URA3::GFP-TUB1/+ SPC42-mcherry::HIS3/+ kar4::KANMX/kar4::URA3 HPH::Pz3ev-IME1/+ leu2::ACT1PR-3ZEV-NatMX/leu2 his3/+ ura3/" met15/+* | S288c |  |
| PJ69-4A | *trp1-901 leu2-3,112 ura3-52 his3-d200 gal4 gal80 GAL2-ADE2 LYS2::GAL1::HIS3 met2::GAL7-lacZ* | S288c | 1 |
| PJ69-4@ | *trp1-901 leu2-3,112 ura3-52 his3-d200 gal4 gal80 GAL-ADE2 LYS2::GAL1::HIS3 met2::GAL7-lacZ* | S288c | 1 |
| MY 16325 | *ura3/" lys2/" ho::LYS2/" arg4-BgIII/+ leu2::HisG/+* | SK1 |  |
| MY 16326 | *ura3/" lys2/" ho::LYS2/" arg4-BgIII/+ leu2::HisG/+ ime4::URA3/"* | SK1 |  |
| MY 16351 | *ho::LYS2/" arg4-BglII/+ +/leu2::hisG lys2-SK1/" ura3::hisG/" kar4::KANMX/+* | SK1 |  |
| MY 16353 | *ho::LYS2/" arg4-BglII/+ +/leu2::hisG lys2-SK1/" ura3::hisG/" slzl::NATMX/"* | SK1 |  |
| MY 16356 | *ho::LYS2/" arg4-BglII/+ +/leu2::hisG lys2-SK1/" ura3::hisG/" kar4::KANMX/" slzl::NATMX/"* | SK1 |  |
| MY 16256 | *leu2/" his3/" ura3/" lys2/+ met15/+ kar4::KANMX/" MUM2-4MYC::HIS3/+ [URA+]* | S288c |  |
| MY 16257 | *leu2/" his3/" ura3/" lys2/+ met15/+ kar4::KANMX/" MUM2-4MYC::HIS3 [KAR4-HA-URA]* | S288c |  |
| MY 16405 | *SLZ1-3HA::HIS3/+ KAR4-9MYC::KANMX/+ kfc1::KANMX/+ his3/" met15/+ leu2/" ura3/" lys2/+* | S288c |  |
| MY 16409 | *SLZ1-3HA::HIS3/+ his3/" met15/+ leu2/" ura3/" lys2/+* | S288c |  |
| MY 13813 | *KANMX::Pzev4-IME1/ime1::KANMX kar4::KANMX/" ura3d52/" his3d100/+ leu2::ACT1PR-42EV-NATMX/leu2 ybr032w::LEU2/+* | S288c |  |
| MY 13815 | *KANMX::Pzev4-IME1/ime1::KANMX kar4::KANMX/+ ura3d52/" his3d100/+ leu2::ACT1PR-42EV-NATMX/leu2 ybr032w::LEU2/+* | S288c |  |
| MY 16456 | *rme1::KANMX/" leu2/" his3/" ura3/" lys2/+ met15/+* | S288c |  |
| MY 16557 | *his3/" leu2/" ura3/" lys2/+ met15/+ rme1(-308A)::KANMX/"* | S288c |  |
| MY 16559 | *his3/" leu2/" ura3/" lys2/+ met15/+ rme1(-308A)::KANMX/" kar4::HPHMX/"* | S288c |  |
| MY 16566 | *leu2/" his3/" ura3/" met15/+ lys2/+ rme1::KANMX/" kar4::HPHMX/"* | S288c |  |
| MY 16550 | *his3/" leu2/" ura3/" lys2/+ met15/+ GFP-IME1/"* | S288c |  |
| MY 16570 | *his3/" leu2/" ura3/" lys2/+ met15/+ GFP-IME1/" kar4::HPHMX/"* | S288c |  |
| MY 16622 | *his3/" leu2/" ura3/" lys2/+ met15/+ rme1Δ::KANMX/" kar4Δ::HPHMX/" GFP-IME1/"* | S288c |  |
| MY 16563 | *leu2/" his3/" ura3/" met15/+ lys2/+ 3xFLAG-RME1/"* | S288c |  |
| MY 16569 | *leu2/" his3/" ura3/" met15/+ lys2/+ 3xFLAG-RME1/" kar4::HPHMX/"* | S288c |  |
| SAy914 | *lys2/" ho::LYS2/" 3xmyc-IME4/"* | SK1 | 2 |
| MY 16543 | *lys2/" ho::LYS2/" 3xmyc-IME4/" kar4::HPHMX/kar4::KANMX* | SK1 |  |
| MY 16616 | *his3/" leu2/leu2::pACT1-Z3EV-natMX +/lys2 met15/+ ura3/"* | S288c |  |
| MY 16532 | *HPHMX::PZ3EV-RIM4/+ leu2::act1pr-Z3EV-NATMX/leu2 ura3/" his3/+ lys2/+* | S288c |  |
| MY 16533 | *HPHMX::PZ3EV-IME1/+ HPH::PZ3EV-RIM4/+ leu2Δ0::act1pr-Z3EV-NATMX/" ura3/"* | S288c |  |
| MY 16534 | *HPHMX::PZ3EV-IME1/+ leu2::act1pr-Z3EV-NATMX/leu2 ura3/" his3/+ lys2/+* | S288c |  |
| MY 16617 | *his3/" leu2/leu2::pACT1-Z3EV-NATMX +/lys2 met15/+ ura3/" kar4::KANMX/"* | S288c |  |
| MY 16531 | *HPHMX::PZ3EV-IME1/+ leu2::act1pr-Z3EV-NATMX/leu2 ura3/" his3/+ met15/+ kar4::KANMX/kar4::URA3* | S288c |  |
| MY 16535 | *HPHMX::PZ3EV-RIM4/+ leu2::act1pr-Z3EV-NATMX/leu2 ura3/" kar4::LEU2/kar4::KANMX4 lys2/+* | S288c |  |
| MY 16536 | *HPHMX::PZ3EV-IME1/+ HPHMX::PZ3EV-RIM4/+ leu2::act1pr-Z3EV-NATMX/" ura3/" kar4::LEU2/kar4::URA3* | S288c |  |
| MY 16619 | *his3/" leu2/leu2::pACT1-Z3EV-NATMX +/lys2 met15/+ ura3/" mum2::KANMX/"* | S288c |  |
| MY 16525 | *HPHMX::PZ3EV-IME1/+ leu2::act1pr-Z3EV-NATMX/leu2 ura3/" lys2/+ his3/+ mum2::URA3/mum2::KANMX* | S288c |  |
| MY 16526 | *HPHMX::PZ3EV-RIM4/+ leu2::act1pr-Z3EV-NATMX/leu2 ura3/" lys2/+ his3/+ mum2::LEU2/mum2::KANMX* | S288c |  |
| MY 16527 | *HPHMX::PZ3EV-IME1/+ HPHMX::PZ3EV-RIM4/+ leu2::act1pr-Z3EV-NATMX/" ura3/" mum2::URA3/mum2::LEU2* | S288c |  |
| MY 16620 | *his3/" leu2/leu2::pACT1-Z3EV-NATMX +/lys2 met15/+ ura3/" slz1::KANMX/"* | S288c |  |
| MY 16537 | *HPHMX::PZ3EV-RIM4/+ leu2::act1pr-Z3EV-NATMX/leu2 ura3/" slz1::LEU2/slz1::KAMNMX his3/+ met15/+* | S288c |  |
| MY 16538 | *HPHMX::PZ3EV-IME1/+ leu2::act1pr-Z3EV-NATMX/leu2 ura3/" slz1::URA3/slz1::KANMX his3/+ lys2/+* | S288c |  |
| MY 16539 | *HPHMX::PZ3EV-IME1/+ HPHMX::PZ3EV-RIM4/+ leu2::act1pr-Z3EV-NATMX/" ura3/" slz1::URA3/slz1::LEU2* | S288c |  |
| MY 16631 | *his3/" leu2/leu2::pACT1-Z3EV-NATMX +/lys2 met15/+ ura3/" ime4::KANMX/"* | S288c |  |
| MY 16434 | *HPHMX::PZ3EV-RIM4/+ leu2::act1pr-Z3EV-NATMX/leu2 ura3/" met15/+ his3/+ ime4::LEU2/ime4::URA3* | S288c |  |
| MY 16435 | *HPHMX::PZ3EV-IME1/+ HPHMX::PZ3EV-RIM4/+ leu2::act1pr-Z3EV-NATMX/" ura3/" ime4::LEU2/ime4::URA3* | S288c |  |
| MY 16313 | *HPHMX::PZ3EV-IME1/+ leu2::act1pr-Z3EV-NATMX/leu2 ura3/" met15/+ his3/+ ime4::LEU2/ ime4::URA3* | S288c |  |
| MY 16621 | *his3/" leu2/leu2::pACT1-Z3EV-NATMX +/lys2 met15/+ ura3/" ime4-cat/"* | S288c |  |
| MY 16615 | *his3/" leu2::pACT1-Z3EV-NATMX/leu2 ura3/" HPHMX::PZ3EV-IME1/+ ime4::LEU2/ime4-cat HPHMX::PZ3EV-RIM4/+* | S288c |  |
| MY 16506 | *HPHMX::PZ3EV-IME1/+ ime4::URA3/ime4-cat his3/+ leu2/leu2::act1pr-Z3EV-NATMX lys2/+ ura3/"* | S288c |  |
| MY 16507 | *his3/+ lys2/+ ura3/" ime4-cat/ime4::LEU2 HPHMX::PZ3EV-RIM4/+ leu2::act1pr-Z3EV-NATMX/leu2* | S288c |  |

References:

1. James P, Halladay J, Craig EA. Genomic libraries and a host strain designed for highly efficient two-hybrid selection in yeast. Genetics. 1996 Dec;144(4):1425-36. doi: 10.1093/genetics/144.4.1425. PubMed PMID: 8978031; PubMed Central PMCID: PMC1207695.
2. Agarwala SD, Blitzblau HG, Hochwagen A, Fink GR. RNA methylation by the MIS complex regulates a cell fate decision in yeast. PLoS Genet. 2012;8(6):e1002732. doi: 10.1371/journal.pgen.1002732. PubMed PMID: 22685417; PubMed Central PMCID: PMC3369947.
