# Supplementary material for: Kar4, the yeast homolog of METTL14, is required for mRNA m6A methylation and meiosis
Source: PLoS Genet. 2023 Aug 21;19(8):e1010896. doi: 10.1371/journal.pgen.1010896 (PMC10470960; doi:10.1371/journal.pgen.1010896)
Supplement: S2 Table — (DOCX) [file pgen.1010896.s008.docx]

| **Table S2. Plasmids used in this paper.** | | |
| --- | --- | --- |
| Strain | Markers | Source |
| pMR 2654 | *CEN ARS URA3 KAR4-3HA* |  |
| pMR 3759 | *GAL4-AD 2µ LEU2 Amp* | 1 |
| pMR 3765 | *GAL4-BD 2µ TRP1 Amp* | 1 |
| pMR 4997 | *KAR4-GBD 2µ TRP1 Amp* | 2 |
| pMR 6455 | *2µ KAR4 URA3 Amp* |  |
| pMR 6895 | *2µ IME1 URA3 Amp* |  |
| pMR 6896 | *2µ IME2 URA3 Amp* |  |
| pMR 6718 | *IME4-GAD 2µ TRP1 Amp* |  |
| pRSM 29 | *Amp KanMx-P4zev* | 3 |
| pAFS 125 | *GFP-TUB1 URA3 Amp* | 4 |
| pRB 3483 | *hphMx-Pz3ev CEN Amp* | 3 |

References:

1. James P, Halladay J, Craig EA. Genomic libraries and a host strain designed for highly efficient two-hybrid selection in yeast. Genetics. 1996 Dec;144(4):1425-36. doi: 10.1093/genetics/144.4.1425. PubMed PMID: 8978031; PubMed Central PMCID: PMC1207695.
2. Lahav R, Gammie A, Tavazoie S, Rose MD. Role of transcription factor Kar4 in regulating downstream events in the *Saccharomyces cerevisiae* pheromone response pathway. Mol Cell Biol. 2007;27(3):818-29. doi: 10.1128/MCB.00439-06. PubMed PMID: 17101777; PubMed Central PMCID: PMCPMC1800688.
3. McIsaac RS, Gibney PA, Chandran SS, Benjamin KR, Botstein D. Synthetic biology tools for programming gene expression without nutritional perturbations in *Saccharomyces cerevisiae*. Nucleic Acids Res. 2014;42(6):e48. doi: 10.1093/nar/gkt1402. PubMed PMID: 24445804; PubMed Central PMCID: PMCPMC3973312.
4. Straight AF, Marshall WF, Sedat JW, Murray AW. Mitosis in living budding yeast: anaphase A but no metaphase plate. Science. 1997;277(5325):574-8. doi: 10.1126/science.277.5325.574. PubMed PMID: 9228009.
